# Screening tools for HIV-associated neurocognitive disorders among adults living with HIV in sub-Saharan Africa: A scoping review

**DOI:** 10.12688/aasopenres.12921.2

**Published:** 2019-10-30

**Authors:** Patrick N. Mwangala, Charles R. Newton, Melanie Abas, Amina Abubakar

**Affiliations:** 1 Centre for Geographic Medicine Research Coast, Kenya Medical Research Institute (KEMRI), Kilifi, P.O BOX 230 - 80108, Kenya; 2 Department of Psychiatry, University of Oxford, Warneford Hospital, Warneford Ln, Oxford OX3 7JX, UK; 3 Department of Public Health, Pwani University, Kilifi, P.O. BOX 195-80108, Kenya; 4 Institute of Psychiatry, Psychology & Neuroscience, King’s College London, 16 De Crespigny Park, London SE5 8AF, UK; 5 Institute for Human Development, Aga Khan University, 2nd Parklands Avenue, Nairobi, P.O. BOX 30270-00100, Kenya

**Keywords:** Screening tools, HIV, HIV-associated neurocognitive disorders, Adults, sub-Saharan Africa, Scoping review

## Abstract

**Background**: People living with HIV are at risk of developing HIV-associated neurocognitive disorders (HAND) which adversely affects their quality of life. Routine screening of HAND in HIV care is recommended to identify clinically important changes in cognitive functioning and allow for early interventions. However, HAND detection in routine clinical practice has never been reported in sub-Saharan Africa (SSA), partly due to a lack of adequately standardized screening tools. This review was conducted to identify the commonly used screening tools for HAND in SSA and document their psychometric properties and diagnostic accuracy.

**Methods:** We searched Ovid Medline, PsycINFO and Web of Sciences databases for empirical studies published from 1/1/1980 to 31/8/2018 on HAND among adults living with HIV in SSA.

**Results:** We identified 14 eligible studies, of which 9 were from South Africa. The International HIV Dementia Scale (IHDS) was the most frequently reported tool, being used in more than half of the studies. However most studies only reported the diagnostic accuracy of this and other tools, with specificity ranging from 37% to 81% and sensitivity ranging from 45% to 100%. Appropriate data on construct validity and reliability of tools was rarely documented. Although most tools performed well in screening for severe forms of HAND, they lacked sensitivity and specificity for mild forms of HAND. NeuroScreen, one of the newer tools, yielded good diagnostic accuracy in its initial evaluation in South Africa (81% to 93% sensitivity and 71% to 81% specificity).

**Conclusions:** This review identified a lack of adequately standardized and contextually relevant HAND screening tools in SSA. Most screening tools for HAND used in SSA possess inadequate psychometric properties and diagnostic accuracy. There is a need for further validation of existing tools and development of new HAND screening tools in SSA.

## Introduction

With the advent of highly active antiretroviral therapy (HAART), the 25 million people living with HIV-1/AIDS in sub-Saharan Africa (SSA) now have the potential for a near-normal life expectancy ^1– 3^. Nonetheless, their wellbeing could be hampered by continuing HIV co-morbidities that adversely affect quality of life. HIV-associated neurocognitive disorder (HAND), at least in its mild form, is one of the commonest comorbidities. Briefly, HAND is a spectrum of neurological complications of HIV infection comprising asymptomatic neurocognitive impairment, mild neurocognitive disorder and HIV-associated dementia ^4^. The diagnosis of HAND is based on the evaluation of key cognitive domains including executive functioning, episodic memory, motor skills, and speed of information processing, language, working memory, and sensory perception according to the Frascati criteria ^4^. These criteria also consider the extent to which the cognitive impairment impacts the person’s performance on activities of daily living.

The prevalence of HAND is estimated to be high across regions, occurring in as many as 50% of all adults living with HIV, including those on HAART and with well-controlled viremia ^5– 7^. Nonetheless, its pattern has changed in the era of HAART ^8^. Notably, the incidence of severe forms of HAND has reduced significantly, while the prevalence of moderate forms has increased ^5– 7^. The persistence of milder forms of HAND is hypothesized to be due to poor adherence to treatment, possible neurotoxicity, multiple co-morbidities, resistance to drugs, low educational achievement, irreversible CNS injury before ART initiation (the so-called legacy effect of untreated HIV), poor CNS penetration of some of the ARV drugs as well as chronic HIV brain infection ^9, 10^.

The magnitude of HAND among adults living with HIV, especially in SSA is largely unrecognized, partly because of the lack of expertise to recognize it and failure to routinely screen for it. Moreover, there is a paucity of well-designed epidemiological studies describing the burden of the condition ^10, 11^. Understanding HAND is important because of its clinical and functional impact on the individual including the heightened risk of mortality ^12^; poor treatment adherence ^13^; poor quality of life ^14^; increased risk-taking behaviors ^15^ and disruptions to everyday functioning ^16^.

Existing guidelines on the management of HAND almost exclusively originate from high-income countries. These guidelines recommend that all people living with HIV be screened for HAND using standardized tools ^17^. Thereafter, the frequency is contingent on whether HAND is already present or whether clinical data suggest increased risk for developing HAND. Additionally, worsening cognitive functioning may necessitate HAART modification when other causes have been excluded ^18^. Other management approaches suggested in the literature include preventative and treatment strategies supporting the biopsychosocial aspects of cognition, such as reducing alcohol and substance use, improving nutrition, treating comorbidities, promoting social contact, reducing depression and stress levels, taking part in cognitively stimulating activities, applying cognitive remediation therapies, and incorporating psychopharmacological interventions ^19, 20^. Nonetheless, there is limited empirical evidence documenting the appropriateness of the suggested HAND management approaches. There is a need for more research to build such evidence and after that, determine which packages of care could potentially be delivered by lay health care workers through task shifting in low-resource settings. Such an approach could prove effective in addressing some of the unique challenges facing the SSA region including inadequate staff with specialized skills, and the many competing healthcare needs.

Despite the potential benefits of early screening, few clinics in SSA screen for this condition in routine HIV care services. This is partly due to the lack of adequately standardized tools of neurocognitive functions ^21^. Another barrier is the prevailing shortage of trained healthcare personnel with expertise to administer these tests.

Information on the most appropriate HAND screening tools in SSA is poorly addressed. In 2013, Zipursky and colleagues conducted the first systematic review of the literature with the aim of evaluating brief screening tools for HAND across the world ^21^. Out of the 31 studies included in the past review, only 5 were from SSA. Overall, the review demonstrated that the commonly utilized screening tools (HIV Dementia Scale (HDS) and the International HIV Dementia Scale (IHDS)) had poor (0.48) and moderate (0.62) pooled sensitivities, respectively. The authors further reported that none of the tools differentiated HAND adequately to suggest wider use. Moreover, substantial methodological flaws were reported in most of the studies. This included: the failure for the studies to use the “gold standard” neuropsychological battery as the reference test, failing to utilize more comprehensive reference tests, non-representativeness of the samples and the varied measurement of functional status. A recently published systematic review provided important information on the comprehensive neuropsychological assessment of NCI in people living with HIV in the SSA ^22^.

Unlike the recently published review which focused on comprehensive neuropsychological assessment in SSA, our scoping review focusses on the HAND screening tools that are being used in the region and document the extent of their validation. Precisely, we document the psychometric properties of these tools. Key constructs of reliability and validity are reviewed. Internal consistency (using Cronbach alpha correlation), test-retest reliability (often measured as correlation, with Pearson *r*), and inter-rater reliability (generally estimated by percent agreement, kappa (for binary outcomes), or Kendall tau) will be examined to describe reliability. For validity, criterion-related validity (predictive, concurrence) and construct validity (convergent, discriminant) ^23^ will be evaluated. The focus on SSA is important to provide context relevant information to guide both clinical and research practice in the region.

## Methods

### Search strategy

A comprehensive database search in Ovid Medline, PsycINFO and Web of Sciences was conducted for peer-reviewed articles published from January 1980 up to 31 ^st^ August 2018. The search strategy was formulated by two reviewers (PNM and AA) and comprised of the following terms combined with Boolean operators: *HIV-associated neurocognitive disorders* OR *cognitive impairment* OR *neurocognitive impairment* OR *neurological complications* AND *HIV* OR *HIV-1* OR *HIV/AIDS* AND *adults* OR *youth* OR *older people* AND *Africa* OR *sub-Saharan Africa*. Additionally, reference lists of retrieved studies were searched for potentially eligible studies that were not identified from the database search.

### Inclusion and exclusion criteria

Studies are eligible if they met the following criteria: i) included participants who were HIV-infected and with documented HAND; ii) focussed on screening HAND; iii) were conducted in SSA; and iv) conducted among adults (mean or median age of at least 18 years). The exclusion criteria are: i) non-empirical studies; ii) studies using other methods for screening HAND apart from brief screening (taking more than 20 minutes); iii) studies published in other languages other than English; iv) studies not conducted among adults, and v) studies carried out outside SSA.

### Data extraction

Data extraction was done by two independent reviewers (PNM and AA). The data was extracted to Microsoft Excel spreadsheets with the following details: first author, date of publication, country of origin, study design, patient characteristics, tool administrators, type of screening tool used, reference neuropsychological tool used, and psychometric properties of the tool. For reliability, we extracted measures of internal consistency, test re-test and inter-rater reliability. For validity, we extracted construct, criterion, divergent or convergent validities whenever reported. Generally, a Cronbach alpha correlation close to 1 is considered good while a Pearson correlation ( r) ≥ 0.70 is considered good ^24, 25^

### Data handling and synthesis

Data analysis involved collating and summarizing of results from individual studies. The synthesis of data extracted from the eligible studies is performed narratively. Frequencies and/or percentages are computed in Microsoft Excel program to summarize the findings on the frequency of the various tools reported in studies. Where applicable, ranges are used to compare the diagnostic accuracy and psychometric properties of the identified HAND screening tools in the final synthesis. Being a scoping review, risk of bias of individual studies was not assessed in this review so as to include as many studies as possible.

## Results

### Study characteristics

**Figure 1.**
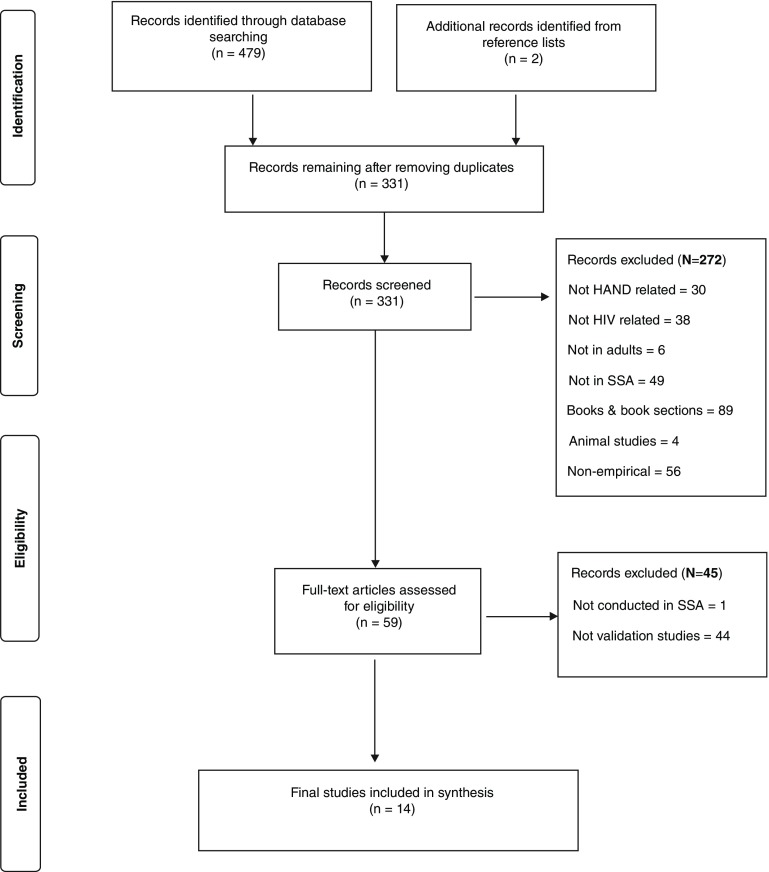
A flow diagram showing the article screening process of this review.

The search strategy yielded 479 studies (flow diagram given in Figure 1). Of the retrieved studies, 14 eligible articles were included in this review, and these were published between 2005 and 2018. All the 14 included studies utilized a cross-sectional design. In total, nine (64.3%) of these studies were conducted in South Africa and the rest were conducted in Nigeria, Uganda and Kenya. The study samples ranged from 16 to 269 participants, as presented in Table 1.

**Table 1.** A summary of the extracted data from eligible studies.

Author	Country & year of publication	Sample size	ART use	Mean/ median age/age range	Tool validated	Reference tool	Issues on HAND diagnosis	Test- retest reliability	Inter-Rater reliability	Sensitivity and Specificity	PPV and NPV	Test administrator	Summary of findings
[ 37] Hakkers	South Africa, 2018	121	All	35	MOCA-B (Montreal cognitive assessment- basic)	NP battery	Used the most recent Frascati criteria to classify HAND). Reference tests were comprehensive (took >90 minutes). However, HAND category was dichotomised into 2 categories only (mild and severe) instead of the 3 standard categories (ANI, MND, HAD). Functional status was not reported	Not done	Not done	40% sensitivity, 72% specificity. Diagnostic accuracy of 59%, Pearson’s r of 0.36.	PPV of 43%, NPV of 70%.	Trained local counsellor	The validity of MOCA-B was poor in this setting. The tool lacked speed of information processing domain. MoCA-B demonstrated best test characteristics when screening for severe forms of HAND.
[ 39] Robbins	South Africa, 2018	102	All	33.31	NeuroScreen	NP battery	Utilized Global Deficit Scores classification to categorize neurocognitive impairment. Reference tests were comprehensive (took >90 minutes). However, functional status was not reported.	Not done	Not done	81% to 93% sensitivity and 71% to 81% specificity for different combination of test scores.	53% to 61% PPV and 92% to 96% NPV.	lay health workers	NeuroScreen is a promising screening tool given the good test characteristics and administration by lay workers. However, the number of false positives is still high (14 to 22 people). More research with larger samples still needed.
[ 33] Gouse	South Africa, 2017	94	75%	37.50	IHDS versus IHDS+HCSQ (International HIV Dementia Scale - HIV Cognitive Symptom Questionnaire)	NP battery	Used the most recent Frascati criteria to categorise HAND into 3 b/groups – ANI, MND and HAD. Reference tests were comprehensive (took >90 minutes). Functional status was not objectively measured.	Not done	Not done	94% sensitivity and 63% specificity for IHDS-HCSQ and 74% sensitivity and 70% specificity for IHDS	74% PPV and 70% NPV for IHDS; 75% PPV and 90% NPV for IHDS-HCSQ	Neuropsyc- ology technician supervised by clinical psychologist.	Supplementing the IHDS with the HCSQ improves its ability to detect severe forms of HAND. The study lacked a control group, did not report construct validity and did not assess feasibility in practice. More studies with large samples needed to evaluate it further.
[ 40] Yechoor	Uganda, 2016	181	80%	37	CogState	NP battery	Utilized Global Deficit Scores classification to categorize neurocognitive impairment. Reference tests were comprehensive (took >90 minutes). However, functional status was not reported.	Not done	Not done	Sensitivity of 57% and specificity of 77% at the optimal cut- off point	60% PPV and 74% NPV at the optimal cut-off	A trained research assistant	CogState may not be a useful tool to screen for HIV-associated NCI in Uganda, as many cases of NCI would be missed.
[ 27] Goodkin	South Africa, 2014	70	NR	31.5	International HIV Dementia Scale	Digit span, TMT	Used the most recent Frascati criteria to categorise HAND into ANI/MND and HAD. Utilized brief reference tests (took <90 minutes). Functional status was not measured hence the merging of ANI/MND.	Not done	Not done	69% Sensitivity, 74% specificity at cut off of 10.5	PPV of 67.7% and NPV of 76.9%	Not reported	The sensitivity for ANI/MND was less than for HAD. Lacked a measure of functional status.
[ 38] Robbins	South Africa, 2013	78	some	29.62	Montreal Cognitive Assessment Test	none	The Study explored the utility of MoCA tool by comparing performance between control and HIV-infected participants. Neurocognitive impairment was not categorized, and neither was functional status measured. No reference tests were used.	Not done	Not done	Not done	Not done	trained research staff	Floor effects were observed on some items. More validation needed.
[ 29] Royal	Nigeria, 2012	116	some	31.7	International HIV Dementia Scale	NP battery	Details on how neurocognitive impairment was classified are not clearly indicated. Reference tests were comprehensive (took >90 minutes). Functional status was measured using the Karnofsky Performance Status.	Not done	Not done	100% sensitivity; 37% specificity at cut off of 10	Not done	Not reported	The tool had very low specificity at the recommended cut off of 10.
[ 31] Kwasa	Kenya, 2012	30	Not reported	39	HIV Dementia Diagnostic Test	NP battery	The study used a slightly modified form of the Frascati criteria to categorize HAND into 3 categories – ANI, MND and HAD. Reference tests were comprehensive (took >90 minutes). Functional status was measured using the Karnofsky Performance Status.	Not done	K = 0.03 – 0.65	63% sensitivity and 67% specificity	Not done	non-physician health workers and experts	Agreement between HCW and expert examiners was poor for many individual items of the tool. The tool had moderate sensitivity and specificity
[ 26] Breuer	South Africa, 2012	269	all	20–42	International HIV Dementia Scale	None	Diagnosis of HAND was made using the IHDS with a score ≤10.5 considered abnormal (positive for dementia). No reference tests were utilized, and neither was functional status measured.	Not done	K of 0.11	50% sensitivity; 69% specificity	Not done	Lay counsellors and nurses	There were many HAD false positives and negatives by counsellors.
[ 28] Joska	South Africa, 2011	190	none	27.5	International HIV Dementia Scale	NP battery	Used the most recent Frascati criteria to categorise HAND into 3 groups – ANI, MND and HAD. Reference tests were comprehensive (took >90 minutes). Functional status was measured using the Patients’ Assessment of Own Function measure.	Not done	Not done	45% Sensitivity, 79% specificity at cut off of 10	Not done	Trained technicians	Individuals with HAD, who screened negative on the IHDS, performed poorly on some tests of executive function.
[ 41] Mupawose	South Africa, 2010	16	none	37.5	Cognitive Linguistic Quick Test	none	Details of how neurocognitive impairment was measured is not included and neither was functional status measured. No reference tests were used.	k of 0.61- 0.90	Not done	Not done	Not done	Trained research assistants	Validation was not done
[ 35] Ogunrin	Nigeria, 2009	240	none	18–64	Modified HIV Dementia Scale	none	Details of how neurocognitive impairment was classified was not reported. The DSM-IV-TR was used as a gold standard. Functional status was assessed using self-report measures.	Not done	Not done	97.3% sensitivity, 80.4% specificity	PPV of 91.4%, NPV of 93.2%	trained research staff	The tool was insensitive to memory impairment in asymptomatic HIV-infected patients.
[ 32] Singh	South Africa, 2008	20	none	34	International HIV Dementia Scale	NP battery	Used the most recent Frascati criteria to categorise HAND. Functional status was not measured.	Not done	Not done	88% Sensitivity; 50% specificity at cut off of 10	Not done	Not reported	IHDS is sensitive, however, its low specificity may limit its clinical utility.
[ 30] Sacktor	Uganda, 2005	181	some	34.2	International HIV Dementia Scale	NP battery	Details of how neurocognitive impairment was classified not reported.	Not done	Not done	80% Sensitivity; 55% specificity at cut off of 10	Not done	Trained physicians	IHDS is sensitive, however, its low specificity may limit its clinical utility

ART, antiretroviral therapy; NPV, negative predictive value; PPV, positive predictive value; NP, neuropsychological battery; NCI, neurocognitive impairment; TMT, trail making test; ANI, asymptomatic neurocognitive impairment; MND, mild neurocognitive disorder; HAD, HIV-associated dementia; HCW, healthcare worker.

### Screening tools identified and their psychometric properties

***IHDS.*** Of the reviewed studies, eight evaluated the validity and diagnostic accuracy of the International HIV dementia scale ^26– 33^ The International HIV dementia scale (IHDS) is a brief HAND screening measure made up of three items assessing motor speed, psychomotor speed and memory specifically developed for use in low- and middle-income countries ^30^. It was initially evaluated in Uganda, where a cut-off of 10 was suggested, yielding a sensitivity of 80% and specificity of 55% ^30^. Subsequent validation studies of the IHDS (included in this review) have reported varied sensitivity and specificity ( Table 1). Overall, sensitivity ranged from 45% to 100% ^28, 29^ and specificity ranged from 37% to 79% ^28, 29^. Data was unavailable on other key components of validity such as test-retest reliability and construct validity.

Kwasa and colleagues ^31^ evaluated the utility of a modified version of the IHDS in Kenya, the HIV Dementia Diagnostic Test, (by adding neurological and functional status items). The modified tool exhibited moderate sensitivity and specificity of 63% and 67% respectively. Nonetheless, the inter-rater reliability was poor (K = 0.03–0.65), with the authors suggesting further training and formal evaluations for health care workers (HCW) to reliably administer the tool. Elsewhere in South Africa, Gouse and colleagues ^33^ evaluated a version of the IHDS which had been modified by adding a brief self-report cognitive tool. The modified IHDS tool was reportedly more effective (94% sensitivity, 63% specificity) in screening for severe forms of HAND than the original IHDS tool (74% sensitivity, 70% specificity).

***HDS.*** HDS was originally developed in 1995 as a brief HAND screening tool ^34^ with four scales designed to screen for cognitive impairments in attention, motor speed, construction and working memory domains. Some of the subtests in the original version of HDS were deemed difficult for administration by non-neurologists and informally trained individuals, which led to the modified version, the IHDS in 2005 ^30^. In the current review, the study by Olubunmi in Nigeria demonstrated good HDS sensitivity (97%) and specificity of 80% with a positive predictive value (PPV) of 91% and a negative predictive value (NPV) of 93% ^35^. Nonetheless, the HDS tool was insensitive to memory impairment in asymptomatic HIV-infected patients. Likewise, data was unavailable on other key components of validity, such as test-retest reliability and construct validity.

***Montreal Cognitive Assessment (MoCA) test.*** The MoCA test is a brief screening tool originally developed in 1996 to screen for mild cognitive impairment ^36^. So far, only two studies in Africa (as identified in this review) have evaluated its utility in screening for HAND, both of which were in South Africa ^37, 38^. In the initial evaluation study ^38^, the tool was able to discriminate neurocognitive performance between HIV-infected and non-infected individuals. However, it has problematic sections that need to be removed. Additionally, a ceiling effect was observed with some of the items making the authors conclude that the tool needs extensive cultural adaptation to make it suitable for South Africa’s linguistically, culturally, educationally, and economically diverse population. Reliability and construct validity were not explored in this initial evaluation. In the second evaluation study ^37^, a moderate correlation (Pearson’s *r* = 0.36) was found between the tool and reference neuropsychological battery. The tool demonstrated a sensitivity of 40%, a specificity of 72% and a diagnostic accuracy of 59%. With these results, the authors concluded that the MoCA test is not a reliable screening tool for cognitive impairment in HIV-infected patients in rural South Africa.

***NeuroScreen.*** NeuroScreen is a computerized neuropsychological screening test battery designed for a smartphone running on the Android operating system. This measure is mobile-based, takes around 15–20 minutes to administer, and needs limited training. It was originally developed and tested in the United States of America ^42^. The measure comprises 10 brief neuropsychological tests assessing verbal learning, memory, processing speed, attention/concentration, executive functioning and motor functioning. In Africa, the NeuroScreen has only been evaluated in South Africa ^39^. In this review, the sensitivity of the NeuroScreen ranged between 81% to 93% and its specificity ranged from 71% to 81% when administered by lay health workers. Unfortunately, data was unavailable on other key aspects of validity such as test-retest reliability and construct validity.

***CogState Brief Battery.*** This is a brief computerized neuropsychological battery (taking around 15 minutes to administer) developed to screen and monitor cognitive impairment in patients including those living with HIV ^43, 44^. In SSA, the tool has been evaluated once in Uganda ^40^. In this evaluation, the tool exhibited a sensitivity of 57% and a specificity of 74% at the optimal cut-off point. Data was also unavailable on other key components of validity such as test-retest reliability and construct validity.

***Cognitive Linguistic Quick Test (CLQT).*** The sixth measure evaluated was the CLQT ^41^. The measure was originally normed on 171 non-clinical and 38 clinical cases in the US as a quick screen for people to identify cognitive strengths and weaknesses in different at-risk populations. In the current review, it is difficult to make conclusions on the potential usefulness of this measure as a screening tool given its small sample size of 16 participants and minimal psychometric evaluations, ^41^.

### Tool administrators

A total of eleven studies described the administrators of the HAND screening tools ^26, 28, 30– 32, 35, 39– 42^. In nine of these studies, the administrators were trained research assistants who were either physicians, neuropsychology technicians, counsellors, nurses or clinical officers. There were two studies that included lay health workers in the screening for neurocognitive impairment ^26, 39^. In one of them, the authors compared the effect of having trained research administrators versus lay administrators ^26^. In the study by Kwasa and colleagues ^31^, the agreement between healthcare workers and expert examiners was poor for many individual items of the tool (K = 0.03–0.6). Similarly, in another study by Breuer and colleagues, lay counsellors tended not to miss symptoms, and detected symptoms more often than nurses for the IHDS ^26^.

### Methodological shortfalls of reviewed studies

Some of the reviewed studies had small sample sizes such as 16 ^41^, 20 ^32^ and 30 ^31^. Additionally, only 9 studies ^28– 33, 37, 39, 40^ utilized comprehensive neuropsychological battery to define HAND in the identified studies. Similarly, only seven studies ^28– 31, 33, 35, 37^ considered the functional status of their participants in categorizing neurocognitive impairment.

## Discussion

We conducted the scoping review to identify the commonly used HAND screening tools in SSA and document their psychometric properties and diagnostic accuracy. The most frequently utilized HAND screening tool identified was the IHDS, observed in more than half of the included studies. The other tools reported were: MoCA, HDS, NeuroScreen, CogState cognitive battery, and the CLQT. Overall, our results show that most of the commonly utilized HAND screening tools in SSA are inadequately validated, with most of the studies only reporting specificity and sensitivity while failing to document important validation constructs notably construct validity and reliability. Besides, close to two-thirds of the included studies originated from a single country (South Africa). This potentially limits the generalizability of the current evidence on screening tools for HAND to other settings in SSA. These results are similar to earlier reviews on HAND screening tools ^21, 45^.

Among the identified tools, IHDS had the most data on validation. However, its validation is suboptimal. Most studies reported only diagnostic accuracy ^26– 32, 35^. There is hardly any data on its construct validity and reliability over time. Besides, the diagnostic accuracy reported is generally not within the expected range (sensitivity of ≥80% and specificity of ≥55%) with some studies having very low specificity (37%) ^29^ and very low sensitivity (45%) ^28^ for the IHDS. The huge variation in the diagnostic accuracy could partly be due to the non-standard definition of HAND in the different studies and possible sociodemographic and clinical heterogeneity of the included samples.

Generally, the limited data on construct validity and reliability as well as the variability of diagnostic accuracy of the reviewed studies do not imply that these measures are less robust and generalizable. This observation calls for more studies to culturally adapt and validate these measures using well-designed studies to better understand their performance in the different settings within the region. With more data, we would be able to accurately determine the psychometric properties of these tools. Typically, a cut-off value of 70% is recommended when selecting an optimal screening tool to yield a demanding threshold for type I and II errors. Besides, screening tools with sensitivity close to a chance level (≤50%) are avoided. In principle, high NPV’s are regarded as important. Going by this guidance, many of the reported indexes of sensitivity, specificity, PPV and NPV would be inadequate. 

NeuroScreen, one of the most recent HAND screening tools is promising. Its initial evaluation in South Africa yielded good diagnostic accuracy when administered by lay healthcare workers. The tool has the potential to address some of the unique challenges and gaps facing resource-limited settings in screening for HAND including difficulty in performing long test batteries, limited screening tools and a shortage of clinical staff. Nonetheless, more research with larger samples should be undertaken to validate the tool in several cultural settings in SSA to clarify its internal and external validity. Some of the issues raised during the initial evaluation need clarification in further studies including the possibility of floor effects and practice effects. The MoCA, the other recent screening test, is gaining momentum in SSA. So far, its validation in SSA has yielded poor criterion validity, showing best test characteristics when screening for severe forms of HAND, before it can be widely used, more studies are needed to validate it.

It is worth noting that several methodological shortfalls were identified in the design and conduct of reviewed studies, which need to be considered by researchers in further validation studies. Some of the studies had small sample sizes and might have lacked the power to detect differences ^31, 32, 44^. There was also a huge variation in the definition of HAND in the studies. Only nine out of the 14 studies reviewed utilized a reference neuropsychological battery to define HAND and few considered the functional consequences brought about by HAND. Additionally, hardly any study reported construct validity and reliability of the screening measure. It is imperative that these aspects of validation are conducted and reported to comprehensively assess the utility of the given tools. 

There is no doubt that the research on HAND screening is gaining momentum in the SSA region, as evidenced by an increasing number of studies on the subject. Nonetheless, there is still a dearth of research on the clinical utility of HAND screening tools. Feasibility data would be critical in guiding the relevant stakeholders make informed decisions regarding the screening of HAND. At the moment, the question as to whether all people living with HIV should be screened for HAND is an ongoing debate ^9^. An expert committee drawn from 30 countries, the Mind Exchange Working Group, recommends the screening of HIV-infected people early in the disease and then every 6–24 months, regardless of symptoms or risk factors ^17^. Another body, the British HIV Association, shares these sentiments by advocating for annual screening without giving details of the methods to be used or which populations to target ^46^. Likewise, the European AIDS Clinical Society recommends that screening should be limited to patients showing symptoms of cognitive impairment ^47^. Generally, universal screening interventions are rarely advocated, except when the screening strategy is very cheap, the screening measure has high accuracy, the consequences for being a false positive (such as distress, anxiety and healthcare costs) are minimal and the consequences of failing to diagnose are grave (that is, there is a highly effective and cost-effective intervention and a very poor outcome without the treatment). One important screening strategy could be to identify known risk factors of HAND (from well-designed epidemiological studies and clinical practice) and use these to develop a risk score and only screen those with a higher risk score (this will increase the diagnostic accuracy of the screening tool). Another strategy could be to train health care workers to be more aware of the signs and cues of HAND and then conduct indicated screening only on patients with suggestive signs and symptoms. 

Our review has the following limitations. Firstly, we did not appraise the quality of the reviewed articles. Nonetheless, this is a primary limitation of scoping reviews; by not considering this, our study considered several articles that would have been excluded in a systematic review. Secondly, it is possible that we could have missed some articles not cited in the target databases during our literature search. Thirdly, the variation in the definition of HAND in some of the included studies may limit the comparison of the screening accuracy of identified tools across studies.

In conclusion, our review shows that there is limited evidence on the reliability and validity of commonly utilized HAND screening tools in SSA. A clear majority of the tools are inadequately validated and standardized with most of the reviewed studies only reporting diagnostic accuracy. Among the studies reporting diagnostic accuracy, there is substantial variability in sensitivity, specificity, PPV, and NPV. However, a few promising tools are available such as the NeuroScreen, but they lack normative data to suggest clinical usefulness. These findings emphasize the need for well-designed studies to culturally adapt and validate HAND screening tools within the region. As this work continues, the next step would be to evaluate the clinical utility of these tools. We acknowledge that this work would require an ongoing effort to correlate tool factors to theorized constructs of HIV disease. In addition to this, tool developers need to bear in mind the unique presentation of certain clients such as those with motor problems who may be naturally biased when taking HAND screening tests with greater emphasis on motor functions. The NeuroScreen presents good progress in this direction by assessing multiple domains of executive functioning, such as working memory and attention. There is also an important need to understand how the current HAND screening tools perform amongst those aging with HIV. So far, none of the studies reviewed has explored this gap in research. 

## Data availability

All data underlying the results are available as part of the article and no additional source data are required.
