# Supplementary material for: Screening tools for HIV-associated neurocognitive disorders among adults living with HIV in sub-Saharan Africa: A scoping review
Source: AAS Open Res. 2019 Oct 30;1:28. Originally published 2018 Nov 19. [Version 2] doi: 10.12688/aasopenres.12921.2 (PMC6914359; doi:10.12688/aasopenres.12921.2)
Supplement: Peer review history

# Peer review: Reviewer response for version 2

**DOI:** 10.21956/aasopenres.14114.r27247

**Published:** 2019-11-14

**Authors:** John A Joska

**Affiliations:** 1 Department of Psychiatry and Mental Health, University of Cape Town, Cape Town, South Africa

I am satisfied that the authors have been responsive to the review.

I confirm that I have read this submission and believe that I have an appropriate level of expertise to confirm that it is of an acceptable scientific standard.

# Peer review: Reviewer response for version 1

**DOI:** 10.21956/aasopenres.13991.r26873

**Published:** 2019-04-24

**Authors:** Anand A. Moodley

**Affiliations:** 1 Department of Neurology, University of the Free State, Bloemfontein, South Africa; 2 Universitas Hospital, Bloemfontein, South Africa

This is a well written and well-presented scoping review on the different screening tools of HAND in SSA. The authors have done an adequate literature search despite their reservations and have presented the currently available data on this very sensitive topic.

 Sadly, there is still no consensus on the definition of HAND which makes comparisons of the different screening tools somewhat superfluous. However, any attempt at formalising the process is a step in the right direction. A useful starting point is a clear definition and rigorously tested diagnostic criteria for HAND. A gold standard using a reference neuropsychological battery is paramount in the definition of HAND. There is a visible void in published studies of a gold standard which thereby diminishes the validity of screening tests.

 As the authors rightfully assert, screening tools for HAND are hardly ever performed due to a lack of standardisation. An essential point that is missing in this review is the difficulty encountered in using the IHDS for patients with hemiparesis of the non-dominant side, quadiparesis and ataxia which are often encountered in HIV infected patients presenting to neurology. Such patients require a test that focuses more on executive functioning rather than motor skills.

 This scoping review sufficiently presents gaps in the knowledge of HAND and its screening. Current screening tests, apart from poor validity also suffer from not being culture-free. In the endeavour to devise a valid and reliable screening test for HAND, clinicians and investigators need to take these various issues into consideration. The test must be valid, reproducible and culture-free to be relevant for use in all SSA countries. The test must be able to differentiate between cortical and subcortical dementia and be relevant in an ageing HIV population.

I confirm that I have read this submission and believe that I have an appropriate level of expertise to confirm that it is of an acceptable scientific standard.

# Response

**Published:** 2019-10-23

**Authors:** patrick Mwangala

**Affiliations:** kemri-wellcome trust research programme, Kenya

We acknowledge the feedback given. Indeed, screening tools e.g. IHDS focussing more on motor skills disadvantage patients presenting with motor difficulties of the limbs. Hence there is a need for tool developers to keep this in mind when developing and refining tools to enhance their utility for such group of patients. Hence, we have noted this in the write-up and inserted the following statement in the conclusion.****

**“…...tool developers also need to bear in mind the unique presentation of certain clients such as those with motor problems who may be naturally biased when taking HAND screening tests with greater emphasis on motor functions. The NeuroScreen presents good progress in this direction by assessing multiple domains of executive functioning such as working memory and attention.**

# Peer review: Reviewer response for version 1

**DOI:** 10.21956/aasopenres.13991.r26745

**Published:** 2019-02-18

**Authors:** John A Joska

**Affiliations:** 1 Department of Psychiatry and Mental Health, University of Cape Town, Cape Town, South Africa

The authors have admirably tackled the thorny issue of screening for HIV-associated neurocognitive disorders (HAND) in Sub-Saharan Africa (SSA). The issue is thorny because the problems of screening span firstly the properties of the tools themselves, and secondly whether or not such tools can be feasibly applied in busy clinical settings. While to some extent, the two problems are linked, the data do not speak directly to the second, and this is where the authors might consider moving these aspects down to a more "discursive" section of the paper, rather than weaving it into the main text.

 The authors are broadly critical of the work done in SSA to date on these measures, primarily on the basis of a lack of evidence of construct validity, and also reliability. They also claim that variability in how HAND was classified across sites, as well as the range of sensitivity and specificity, limit how robust and generalisable the measures are. In lesser part, they are critical of the possibility that studies are limited in power, and by the application of gold standard test batteries.

 In my view, not all of these criticisms are correct or fair. It is true that #construct_validity has not been properly addressed by researchers. It must be borne in mind that this requires an ongoing effort to correlate tool factors to theorised constructs of the underlying disease/disorder. In the case of a tool such as the IHDS, each of the 3 sub-items is based on existing neuropsychological sub-tests, so possibly at least some elements of #construct_validity are dealt with. It might be possible to have conducted some type of factor analysis or matrix analysis on data from authors.

 The problem of how HAND is assessed across sites was not presented in the main Table, so it is not possible to evaluate whether this was in fact the case or not. The authors also state that "only" 9/14 papers utilised detailed neuropsychological batteries. I venture that 65% is not a minority but a reasonable proportion. 

 Other specific comments to consider:

- In the Abstract, the word "subtle" is used to describe the type of changes intended for HAND screening. I don't believe that this is the intention of any tool. Rather, most clinicians would be keen to identify severe, or clinically symptomatic disease.
- It is also noted in the Abstract that HAND screening is rarely done in SSA- but I suspect it is almost never done in ANY routine clinical practice. At least there are NO publications that I know of that report this.
- In the Introduction, the words "moderate" and "milder" are used interchangeably. While the context is mainly clear to an informed reader, it can be confusing. I suggest that the authors stick to one use of the construct of "less severe".
- Later in para 2 of the Introduction, possible causes of cognitive impairment in HIV are mentioned. But two of the most prevalent possibilities are not: namely low education, and the legacy effect of untreated HIV.
- In the 4th para, a reference is made to "adults at risk" of HAND but is only dealt with in passing. This is a hugely contentious topic. There are some data to suggest that low CD4 nadir, low education and some other factors are risk factors, but it is unclear whether this is of any help in SSA, when low CD4 nadir and low levels of education have historically been the norm. Perhaps either remove this reference, or use a separate paragraph to describe what it might be used for, then move on.
- The authors mention that a "package" of interventions for patients with HAND might be transferable by task-sharing to lay health workers. I would strongly suggest that many of these listed skills and interventions would not be. And that only a small number would be implementable by a LHW.
- Would the listing of the statistical parameters needed for good quality studies not be better mentioned in the Methods section?
- The issue of feasibility is not really addressed in the Introduction, and as there are no data in the Results that speak to it, makes my point above. This issue is perhaps beyond the "scope" of this review, and could be included as a small discursive paragraph.
- This paper did not seem to make it in for some reason: Comparison of Five Brief Screening Tools for HIV-Associated Neurocognitive Disorders in the USA and South Africa. Joska JA, Witten J, Thomas KG, Robertson C, Casson-Crook M, Roosa H, Creighton J, Lyons J, McArthur J, Sacktor NC. AIDS Behav. 2016 Aug;20(8):1621-31 ^1^.
- In the Discussion, the "variable" sensitivity and specificity are cited as a reason that the tools are sub-optimal. If the psychometric properties of the tools are at stake, then more detailed discussion of "decent" sensitivity/specificity needs to be included, with a discussion of NPV and PPV. Variability on its own is not an issue?
- Para 4 of the Discussion goes into some length about the feasibility of screening. As this review does not obtain data on any of these parameters, it is probably advisable to drop this to a lower discursive commentary. In the same para, the authors refer to the idea that the screening tools should be subject to "interventional research"- can this be clarified, as it could mean different things eg. interventions for HAND, or interventions to ascertain construct validity. Also, the term "clinical utility" is mentioned, followed by "cost-effectiveness" in parentheses. This needs to be clarified. This is again referred to in related to cost-effectiveness. If cost-effectiveness is a major need, then perhaps some detail on how this might be done should be supplied.

I confirm that I have read this submission and believe that I have an appropriate level of expertise to confirm that it is of an acceptable scientific standard, however I have significant reservations, as outlined above.

## References

[1] : A Comparison of Five Brief Screening Tools for HIV-Associated Neurocognitive Disorders in the USA and South Africa. *AIDS Behav*.20(8) : 10.1007/s10461-016-1316-y 1621-31 10.1007/s10461-016-1316-y 26860536PMC5771655

# Response

**Published:** 2019-10-23

**Authors:** patrick Mwangala

**Affiliations:** kemri-wellcome trust research programme, Kenya

The authors have admirably tackled the thorny issue of screening for HIV-associated neurocognitive disorders (HAND) in Sub-Saharan Africa (SSA). The issue is thorny because the problems of screening span firstly the properties of the tools themselves, and secondly whether or not such tools can be feasibly applied in busy clinical settings. While to some extent, the two problems are linked, the data do not speak directly to the second, and this is where the authors might consider moving these aspects down to a more "discursive" section of the paper, rather than weaving it into the main text.  

***Response:***

***As suggested by the reviewer, the paragraph highlighting the clinical utility of HAND screening tools has been moved down, towards the end of the discussion.***

 The authors are broadly critical of the work done in SSA to date on these measures, primarily on the basis of a lack of evidence of construct validity, and also reliability. They also claim that variability in how HAND was classified across sites, as well as the range of sensitivity and specificity, limit how robust and generalizable the measures are. In lesser part, they are critical of the possibility that studies are limited in power, and by the application of gold standard test batteries.

 In my view, not all of these criticisms are correct or fair. It is true that #construct validity has not been properly addressed by researchers. It must be borne in mind that this requires an ongoing effort to correlate tool factors to theorized constructs of the underlying disease/disorder. In the case of a tool such as the IHDS, each of the 3 sub-items is based on existing neuropsychological sub-tests, so possibly at least some elements of #construct validity are dealt with. It might be possible to have conducted some type of factor analysis or matrix analysis on data from authors.

***Response***

***We share the reviewer’s sentiments regarding the validity of the commonly used screening tools in the region up to date. Our arguments don’t in any way disregard the utility of the tools in question especially in the context of research. Our findings majorly emphasize the need for more focussed studies evaluating the construct validity and reliability of these tools (which is inadequately addressed at the moment) while at the same time appreciating the progress made so far. We have exercised due diligence in highlighting this observation in the revised document.***

***The suggested analyses (factor analysis or matrix analysis) would be difficult to do in the given set of results. This partly because of the heterogeneity of the included studies. Besides, we would require the original datasets of the studies which is beyond the scope of this review.***

 The problem of how HAND is assessed across sites was not presented in the main Table, so it is not possible to evaluate whether this was, in fact, the case or not. The authors also state that "only" 9/14papers utilized detailed neuropsychological batteries. I venture that 65% is not a minority but a reasonable proportion.

***Response***

***A column has been added in the main Table highlighting how HAND was assessed across the included studies. Indeed, 65% (proportion of the studies which used neuropsychological batteries in assessing HAND) is a reasonable figure. However, this figure should not be viewed in isolation when evaluating how HAND was assessed. Some of the studies (in the 65% figure) did not assess the functional status of the individuals while some did not categorize HAND into the 3 categories recommended by the Frascati criteria. Such methodological differences make it difficult to accurately compare such studies.***

 Other specific comments to consider:

 In the Abstract, the word "subtle" is used to describe the type of changes intended for HAND screening. I don't believe that this is the intention of any tool. Rather, most clinicians would be keen to identify , or clinically symptomatic disease.

**Respone**

***This has been revised to reflect ‘clinically important changes’ and has been updated accordingly in the abstract.***

 It is also noted in the Abstract that HAND screening is rarely done in SSA- but I suspect it is almost never done in ANY routine clinical practice. At least there are NO publications that I know of that report this.

***Response***

***We agree with the reviewer. To our knowledge, the screening of HAND in routine clinical practice has never been reported in any setting in the SSA region. This has been adjusted accordingly.***

 In the Introduction, the words "moderate" and "milder" are used interchangeably. While the context is mainly clear to an informed reader, it can be confusing. I suggest that the authors stick to one use of the construct of "less severe".

***Response***

***We agree with the reviewer. For clarity and consistency purposes, we have maintained the term ‘mild’ and dropped ‘moderate’ whenever we have used the term to imply ‘less severe’ in to HAND*** *.*

 Later in para 2 of the Introduction, possible causes of cognitive impairment in HIV are mentioned. But two of the most prevalent possibilities are not: namely low and the legacy effect of untreated HIV.

***Response***

***This has been noted. We have included these factors as some of the possible causes of HIV-associated neurocognitive impairment in the second paragraph of the introduction.***

 In the 4th para, a reference is made to "adults at risk" of HAND but is only dealt with in passing. This is a hugely contentious topic. There are some data to suggest that low CD4 nadir, low education some other factors are risk factors, but it is unclear whether this is of any help in when low CD4 nadir and low levels of education have historically been the norm. Perhaps either remove this reference or use a separate paragraph to describe what it might be used for, then move on.

***Response***

***We agree with the reviewer that this is a contentious issue. While there is some data suggesting that certain factors e.g. low CD4 nadir, could be risk factors generally, there is limited evidence*** ***from well-designed epidemiological studies within the region documenting such factors. We have deleted this reference in the revised version of the writeup.***

 The authors mention that a "package" of interventions for patients with HAND might be transferable by task-sharing to lay health workers. I would strongly suggest that many of these listed skills and interventions would not be. And that only a small number would be implementable by .

***Response***

***We agree with the reviewer, and this has been revised accordingly. We have inserted the following statement.***

***“……there is limited empirical evidence documenting the appropriateness of the suggested HAND management approaches. There is a for more research to build such evidence and thereafter*** ***determine which packages of care could potentially be delivered by lay health care workers through task shifting in low-resource settings. Such an approach could prove effective in addressing*** ***some of the unique challenges facing the SSA region including inadequate staff with specialized skills, and the many competing healthcare needs.”***

 Would the listing of the statistical parameters needed for good quality studies not be better mentioned in the Methods section?

***Response***

***We agree with the reviewer’s suggestion. We have moved the portion describing statistical parameters needed for good quality studies to the methods section of the paper under ‘Data*** ***extraction.’***

 The issue of feasibility is not really addressed in the Introduction, and as there are no data in the Results that speak to it, makes my point above. This issue is perhaps beyond the "scope" of this and could be included as a small discursive paragraph.

***Response***

***We agree with the reviewer’s observation and as earlier stated, we have moved this portion to a smaller discursive part towards the end of the paper.***

 This paper did not seem to make it in for some reason: Comparison of Five Brief Screening Tools Joska JA, Witten J HIV-Associated Neurocognitive Disorders in the USA and South Africa KG, Robertson C, Casson-Crook M, Roosa H, Creighton J, Lyons J, McArthur J, SacktorNC. AIDS Behav. 2016 Aug;20(8):1621-31.

***Response***

***The cited paper was excluded from the analysis because it did not aggregate the relevant results (psychometric properties of the different tools) by geographic location; USA versus South*** ***Africa. All the relevant results presented were combined results of the two settings.***

 In the Discussion, the "variable" sensitivity and specificity are cited as a reason that the tools are sub-optimal. If the psychometric properties of the tools are at stake, then detailed discussion of "decent" sensitivity/specificity needs to be included, with a discussion of NPV and PPV. Variability on its own is not an issue?

***Response***

*From the results of this paper, we cannot make the conclusion the tools observed are ‘suboptimal’, hence not fit for use. Our key observation and finding that virtually all studies reviewed hardly reported construct validity and reliability of the tools they used. Besides, we also note that there is variability in the reported indexes of sensitivity, specificity, PPV, NPV. We have included the following statement (third paragraph of the discussion).*

***“Generally, the limited data on construct validity and reliability as well as the variability of diagnostic accuracy of the reviewed studies do not imply that these measures are less robust and . This observation calls for more studies to culturally adapt and validate these measures using well-designed studies to better understand their performance in the different settings within the region. With more data, we would be able to accurately determine the psychometric properties of these tools and determine which ones are consistently performing poorly and well. Normally, a cut-off value of 70% is recommended when selecting an optimal screening tool to yield a demanding threshold for type I and II errors. Besides, screening tools with sensitivity close to a chance level (≤50%) are avoided. In principle, high NPV’s are regarded as important. Going by , many of the reported indexes of sensitivity, specificity, PPV NPV would be inadequate.”***

 Para 4 of the Discussion goes into some length about the feasibility of screening. As this review does not obtain data on any of these parameters, it is probably advisable to drop this to a lower discursive commentary. In the same para, the authors refer to the idea that the screening tools should be subject to "interventional research"- can this be clarified, as it could mean different things e.g interventions for HAND, or interventions to ascertain construct validity. Also, the term "clinical utility" is mentioned, followed by "cost-effectiveness" in parentheses. This needs to be clarified. This is again referred to in related to cost-effectiveness. If cost-effectiveness is a major need, then perhaps some detail on how this might be done should be supplied.

***Response***

***This has been noted, and in response, we have reduced and shifted this portion to a less discursive portion towards the end of the discussion. The small section on interventional research when evaluating the clinical utility of HAND screening tools has been dropped as the data do not directly speak to this.***
